# CyTOF Profiling of Zika and Dengue Virus-Infected Human Peripheral Blood Mononuclear Cells Identifies Phenotypic Signatures of Monotype Subsets and Upregulation of the Interferon-Inducible Protein CD169

**DOI:** 10.1128/mSphere.00505-21

**Published:** 2021-06-23

**Authors:** Rafael Fenutria, Kevin Maringer, Uma Potla, Dabeiba Bernal-Rubio, Matthew J. Evans, Eva Harris, Adeeb H. Rahman, Ana Fernandez-Sesma, Irene Ramos

**Affiliations:** a Department of Microbiology, Icahn School of Medicine at Mount Sinai, New York, New York, USA; b Division of Infectious Diseases and Vaccinology, School of Public Health, University of California, Berkeley, California, USA; c Human Immune Monitoring Center, Icahn School of Medicine at Mt. Sinai, New York, New York, USA; d Department of Genetics and Genomic Sciences, Icahn School of Medicine at Mt. Sinai, New York, New York, USA; e The Graduate School of Biomedical Sciences at Icahn School of Medicine at Mount Sinai, New York, New York, USA; University of Texas Southwestern Medical Center

**Keywords:** dengue virus, *ex vivo*, immune profiling, mass cytometry, myeloid cells, peripheral blood mononuclear cells, Zika virus

## Abstract

Zika and dengue virus (ZIKV and DENV) are two flaviviruses responsible for important vector-borne emerging infectious diseases. While there have been multiple DENV epidemics in the last decades, there have been fewer documented epidemics caused by ZIKV until recent years. Thus, our current knowledge about the biology of ZIKV, the disease, and the immune responses in humans is limited. Here, we used mass cytometry (CyTOF) to perform a detailed characterization of the innate immune responses elicited by ZIKV and DENV in human peripheral blood mononuclear cells (PBMCs) from healthy donors infected *ex vivo*. We found that ZIKV and DENV exposure of human PBMCs induces global phenotypic changes in myeloid cells, characterized mainly by upregulation of costimulatory molecules (CD86 and CD40), CD38, and the type I interferon-inducible protein CD169, a marker for phagocytic function and cross-priming potential in myeloid cells. We also found that ZIKV induces expansion of nonclassical monocytes in cell culture. The analysis of the phenotype of the three monocyte subtypes (classical, intermediate, and nonclassical) at the single-cell level identified differences in their expression of CD86, CD38, CXCL8, and CXCL10 during ZIKV and DENV infection. Overall, using CyTOF, we found that *ex vivo* infections of PBMCs with ZIKV and DENV reproduced many aspects of the profile found in blood from patients in previously described cohort studies, which highlights the suitability of this system for the study of the human host responses to these viruses.

**IMPORTANCE** Zika and dengue viruses are emergent arboviruses of great public health impact. Both viruses are responsible for important diseases, yet there is currently no vaccine or specific treatment available. Immune cells play critical roles in the virus cycle as well as in the innate and adaptive immune response elicited in the host; therefore, it is critical to understand the changes induced by virus infection in peripheral blood mononuclear cells (PBMCs). In this study, we used a model of *ex vivo* infection of PBMCs and CyTOF technology to profile the early innate immune changes induced by Zika virus and dengue virus in blood.

## INTRODUCTION

Zika virus (ZIKV) and dengue virus (DENV) belong to the family *Flaviviridae* and are emergent arboviruses of great public health impact (1). ZIKV was first isolated in the Zika Forest in Uganda in 1947 (2). However, it was not until 2007 when the first outbreak in humans occurred (3). In 2015, a ZIKV epidemic started in Brazil, and the virus spread rapidly to 48 countries in the American continent in less than 2 years. ZIKV generally causes mild, self-limiting febrile disease in humans, and a large number infections are asymptomatic. However, multiple cases of a congenital syndrome associated with ZIKV infection were reported (4). The mechanisms behind the sudden rapid spread of the virus in the human population are not clear and continue under investigation (5). Since ZIKV has not caused significant disease in humans until recently, the disease, pathogenesis, and specific innate immune responses elicited against this virus upon infection are not well understood.

Infections by DENV also represent a major public health concern given the increasing number of cases and spread geographic distribution in the last decades (6). Most of the DENV infections are believed to be asymptomatic based on modeling studies (7). Common symptoms associated with DENV infection in symptomatic patients or dengue fever (often self-limiting) are fever, headache, myalgia, joint pain, and lesions on the skin. Complications occur in some cases, known as severe dengue, and are characterized by severe plasma leakage and, in some cases, hemorrhage and/or organ failure (8). There are four serotypes of DENV, namely, DENV1, DENV2, DENV3, and DENV4. While most primary DENV infections are mild or subclinical, a secondary infection with a different serotype can lead to severe dengue due to antibody-dependent enhancement (ADE), among other factors (9).

Both ZIKV and DENV are introduced into the skin by a mosquito bite. ZIKV has been shown to infect cells that are present in the skin, such as fibroblasts, keratinocytes, and dendritic cells (DC) (10), while DENV infects antigen-presenting cells (either resident or recruited to the skin) such as DCs or macrophages (11, 12).

Upon virus internalization, DCs migrate to the lymph nodes to present antigen to T cells and B cells initiating the adaptive immune response. Importantly, innate immune cells such as DCs, macrophages, or monocytes are highly proficient at capturing antigens given the elevated levels of surface receptors such as C-type lectins (13, 14) and high levels of pattern recognition receptors (PRRs) such as RIG-I like receptors (RLR) or toll-like receptors (TLRs) (15). Interestingly, both ZIKV and DENV have been found to infect blood monocytes in infected patients (11, 16–19). *Ex vivo* infection of peripheral blood mononuclear cells (PBMCs) allows us to study innate immune responses induced by virus infection at early times after infection, which are usually difficult to capture *in vivo*. Here, we applied mass cytometry (CyTOF) technology to analyze the innate immune profile induced in immune cells by ZIKV and DENV.

## RESULTS

### ZIKV and DENV2 exposure of human PBMCs induces phenotypic changes in myeloid antigen-presenting cells (APC).

**FIG 1:**
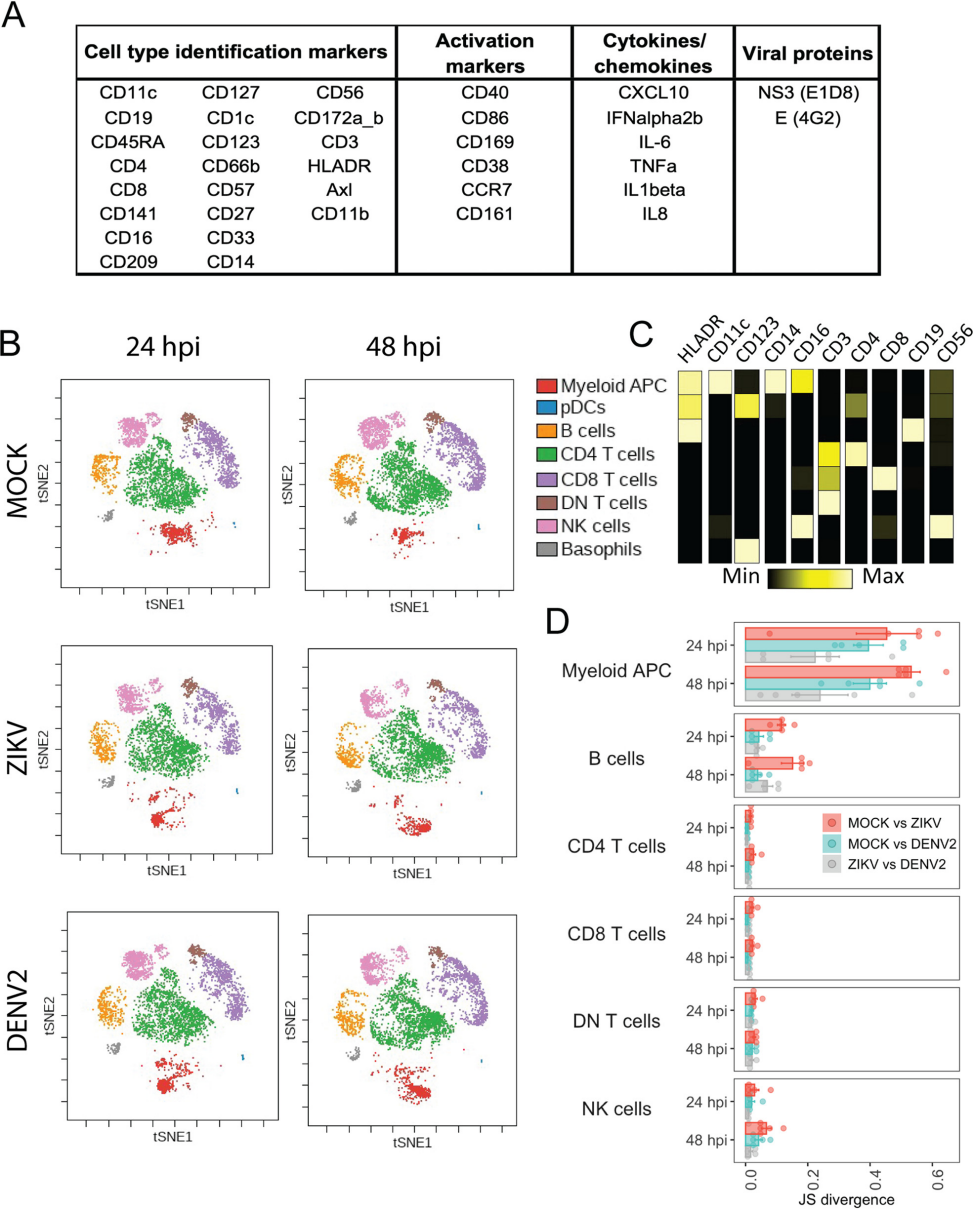
Immune phenotyping of mock, ZIKV, and DENV2 *ex vivo*-infected PBMCs using CyTOF. (A) Panel of markers used for sample preparation. (B) viSNE plots of PBMCs from a representative donor exposed to ZIKV, DENV2, or mock treatment, indicating main cell populations. (C) Median marker intensity (for main cell identification markers) per cell population. Names of the identified populations are indicated as part of the legend in panel B. (D) Pairwise Jensen-Shannon (JS) divergence calculated for viSNE across cells from 5 donors exposed to ZIKV, DENV2, or mock treatment. pDCs and basophils were excluded of this analysis given the low representation of those cells in some viSNE visualizations.

In order to profile the phenotype that ZIKV and DENV2 induce in human immune cells, we applied the CyTOF technology to PMBCs after exposure to these viruses. Blood from five independent donors was obtained, and PBMCs were infected at a multiplicity of infection (MOI) of 1 for 24 or 48 h. As shown in Fig. 1A, we used a total of 36 markers for this analysis, including surface phenotypic markers for cell identification, activation markers, intracellular cytokine staining, and virus protein markers. We first used viSNE (20) analysis to visualize global changes in the phenotypic landscape of the cells exposed to the viruses. Since we were interested in phenotypic changes, viral proteins were not included as clustering markers (eliminating possible clustering driven by virus presence).

Figures 1B and C show the main populations that were identified using cell type identification markers in the viSNE visualizations. This analysis revealed that the phenotype of myeloid APC, identified by their high expression of CD11c and HLA-DR, was strongly modified after infection with ZIKV or DENV2 compared to mock infection. To quantify the divergence across populations in the two-dimensional maps from the viSNE visualization, we used the Jensen-Shannon (JS) divergence (20), which measures the similarity between two probability distributions. Specifically, we performed pairwise comparisons of the treatments (mock versus ZIKV, mock versus DENV2, and ZIKV versus DENV2) of the main populations identified in the viSNE plots for PBMCs for each of the 5 donors (Fig. 1D). Again, the population of myeloid APC showed the highest divergence levels between mock- and ZIKV- or DENV2-infected PBMCs at both 24 and 48 h postinfection (hpi). JS divergence between ZIKV- and mock-infected cultures was higher than that between DENV2- and mock-infected cultures in the myeloid APC population. Population of B cells also showed some divergence between mock- and ZIKV-infected cells. However, the global phenotype of CD4 T cells, CD8 T cells, double-negative (DN) T cells, or NK cells was not affected by infection, as indicated by the low level of JS divergence when different conditions were compared (Fig. 1B and D).

### ZIKV and DENV2 infect myeloid APC within PBMCs in culture and induce upregulation of their activation markers.

**FIG 2:**
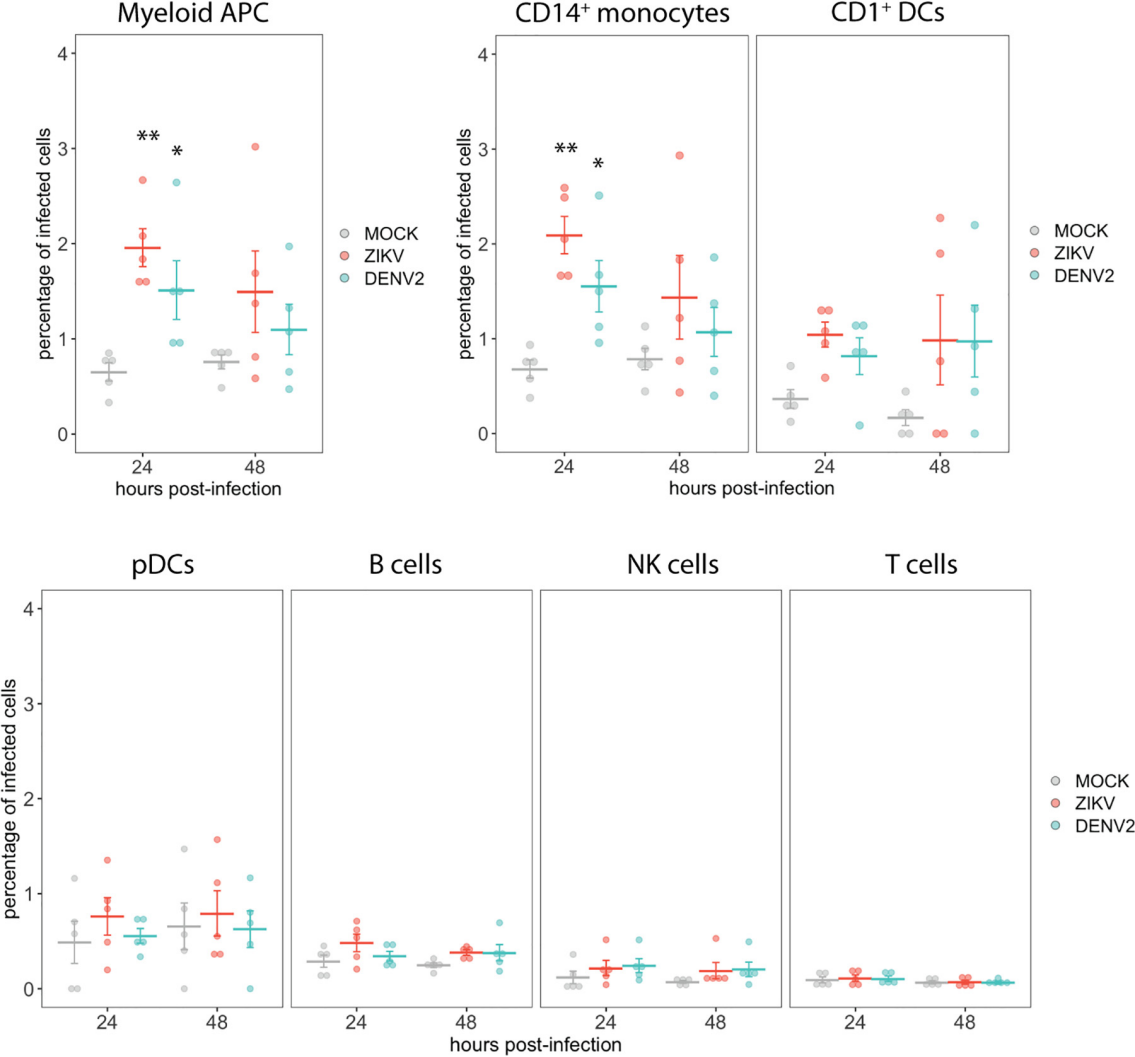
CyTOF analysis of the percentage of infected cells in different cell populations after mock, ZIKV, or DENV2 exposure. The percentage of cells with expression of the viral NS3 and E (double stained with E1D8 and 4G2 antibodies) were determined within the main populations of PBMCs after mock, ZIKV, or DENV2 *ex vivo* exposure of PBMCs from 5 healthy donors. Means ± standard errors of the means (SEM) are indicated. **, *P* < 0.01; *, *P* < 0.05.

The changes in the phenotypic landscape detected by the viSNE analysis in ZIKV-exposed samples compared to mock treatment suggest uptake and sensing of the virus by cells present in the PBMCs. Therefore, we next evaluated the levels of infection by quantifying the percentage of cells expressing both viral E protein and the nonstructural protein 3 (NS3) within different populations of PBMCs (gating strategy shown in Fig. S1 and S2 in the supplemental material). Since NS3 is a nonstructural viral protein, its expression is indicative of virus replication within cells. As shown Fig. 2, we detected infected cells in the myeloid APC population at 24 hpi in ZIKV- and DENV2-exposed cultures. Within the myeloid APC population, we also analyzed the levels of infection in CD14^+^ monocytes and CD1c^+^ DCs, and we found a significant detection of infected cells in the population of CD14^+^ monocytes at 24 hpi. We also detected some levels of infection of CD1c^+^ DC populations in some subjects; however, we did not observe a significant statistical increase in the percentage of infected cells in this population when data from 5 independent donors were analyzed. In the case of the other populations, such as plasmacytoid DCs (pDCs), B cells, T cells, and NK cells (all of them belonging to the lymphoid lineage), we did not detect cells expressing E and/or NS3 viral proteins (Fig. 2).

10.1128/mSphere.00505-21.1 FIG S1 Gating strategy for dendritic cells, myeloid cells, and monocytes. Download FIG S1, PDF file, 0.4 MB. Copyright © 2021 Fenutria et al. 2021 Fenutria et al. https://creativecommons.org/licenses/by/4.0/ This content is distributed under the terms of the Creative Commons Attribution 4.0 International license.

10.1128/mSphere.00505-21.2 FIG S2 Gating strategy for T cells, B cells, and NK cells. Download FIG S2, PDF file, 0.2 MB. Copyright © 2021 Fenutria et al. 2021 Fenutria et al. https://creativecommons.org/licenses/by/4.0/ This content is distributed under the terms of the Creative Commons Attribution 4.0 International license.

**FIG 3:**
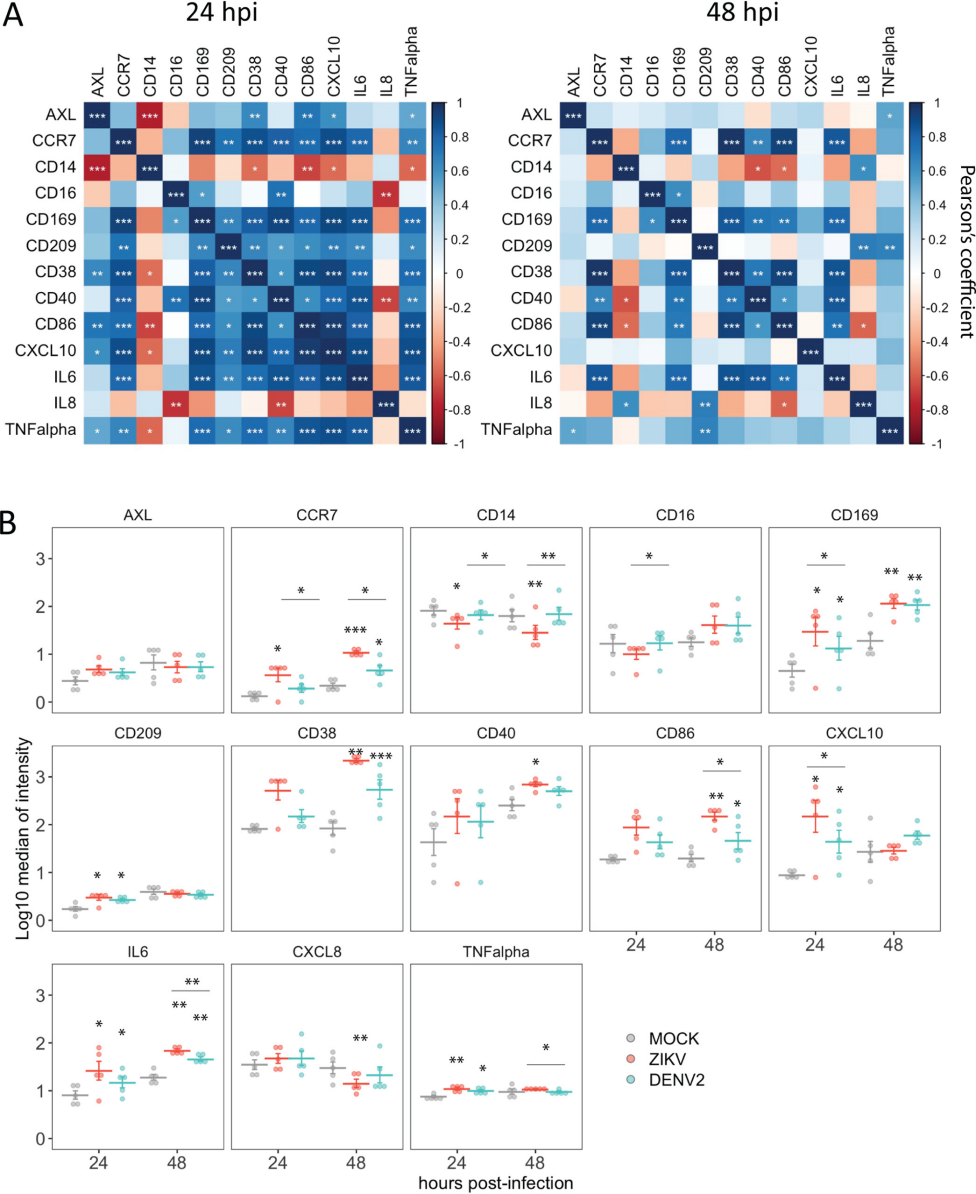
Expression of selected activation markers, cytokines, and chemokines in myeloid APC (HLADR^+^ CD11c^+^). (A) Pearson’s correlation among markers. (B) Quantification of the expression of those markers in mock-, ZIKV-, or DENV2-exposed PBMC samples from 5 healthy donors. Means ± SEM are indicated. Stars without bars in panel B indicate statistically significant differences with mock treatment. ***, *P* < 0.001; **, *P* < 0.01; *, *P* < 0.05.

As we found dramatic changes in the phenotype of myeloid APC after exposure to ZIKV as well as some level of infection, we studied this population (CD11c^+^, HLADR^+^) in more detail. First, we selected the most relevant markers for immune functions of myeloid APC from the panel, including costimulatory molecules, adhesion molecules, cytokines, and chemokines. We then explored the relationship among the expression (median of intensity) of these markers in all the samples included in the study (5 donors). To account for phenotypic differences that could arise over time during the cell culture as a consequence of cells being removed from their physiological environment, we performed all the comparisons across treatments within the same time point. As expected, we found a positive correlation (Pearson’s method) among most of them, which was highly significant at 24 hpi in most cases (Fig. 3A). However, we found that the CD14 receptor showed a negative correlation with most of the other markers, which was significant at 24 hpi in the case of CD38, CD86, CXCL10, and AXL. Next, we analyzed the changes in the expression of those markers in ZIKV- and DENV2-infected samples compared to mock treatment at 24 and 48 hpi. As shown in Fig. 3B, we found upregulation of CCR7, CD169, CD209, CD38, CD86, CXCL10, interleukin-6 (IL-6), and tumor necrosis factor alpha (TNF-α) at 24 hpi, 48 hpi, or both time points in ZIKV- and DENV2-exposed cultures. However, we observed a significant decrease in the expression of CD14 in ZIKV-infected cells at both time points compared to mock- and DENV2-infected cells. Therefore, exposure of PBMCs to ZIKV and DENV results in an increase in the expression of activation markers and cytokines of myeloid APC, and a decrease in the expression of the receptor CD14 was found in ZIKV-exposed cultures.

### ZIKV and DENV2 infections modify the phenotypic profile of blood monocytes.

**FIG 4:**
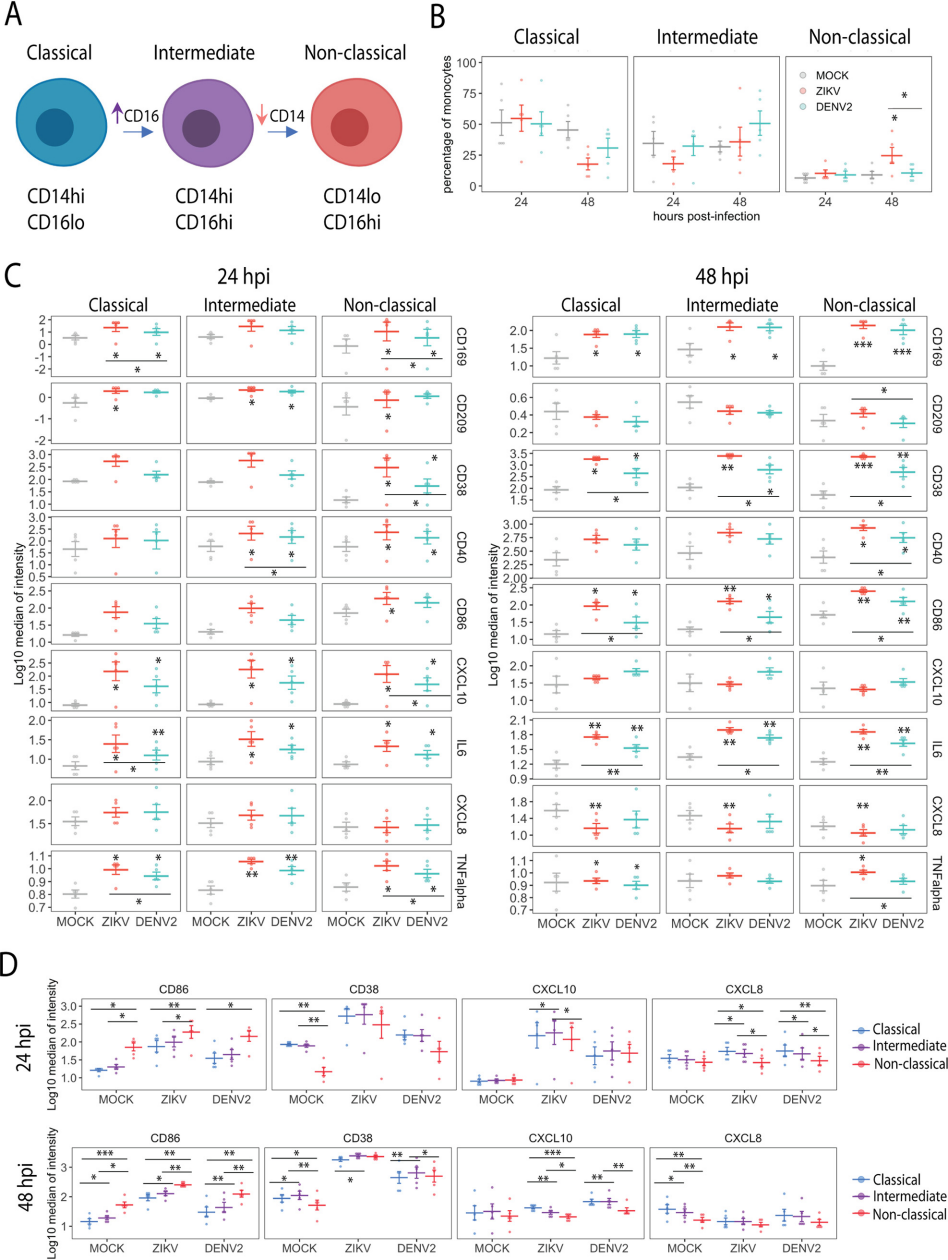
CyTOF analysis of monocyte populations in mock, ZIKV, or DENV2 *ex vivo*-exposed PBMCs. (A) Scheme of the subsets and differentiation stages of circulating monocytes. (B) Frequencies of monocyte subsets in the monocyte compartment (HLADR^+^ CD1c^−^ CD141lo CD11c^+^). (C) Quantification of selected activation markers and cytokines in classical, nonclassical, and intermediate monocytes upon infection with ZIKV or DENV2. (D) Comparison of selected activation markers and cytokines among the different monocyte subtypes at 24 and 48 hpi. Data from cells from 5 donors are shown. Means ± SEM are indicated. Stars without bars in panels B, C, and D indicate statistically significant differences with mock treatment. ***, *P* < 0.001; **, *P* < 0.01; *, *P* < 0.05.

The expression of CD14 and CD16 in monocytes define three subtypes of blood monocytes, namely, classical (CD14hi CD16lo), intermediate (CD14hi CD16hi), and nonclassical (CD14lo CD16hi) (21), and changes in the proportion of these monocyte subtypes have been identified in blood from ZIKV and DENV patients (17, 19, 22). Therefore, since we found changes in the expression of CD14 in ZIKV-exposed cultures, we analyzed the monocyte subtypes in detail. It has been reported that these three subtypes represent different stages of differentiation, transitioning from classical to intermediate subtype through upregulation of CD16, and from intermediate to nonclassical phenotype through downregulation of CD14 (23), as depicted in Fig. 4A. It is also known that the frequencies of these monocyte subtypes vary in certain conditions, such as viral infections, bacterial infections, and autoimmune disorders (24). Therefore, we quantified the percentage of each type of monocytes within the total monocyte compartment (CD11c^+^ cells excluding CD141^+^ and CD1c^+^ DCs) in infected and uninfected cultures. As shown in Fig. 4B, a significant increase of the proportion of nonclassical monocytes was detected in samples exposed to ZIKV at 48 hpi. A trend toward higher levels of intermediate monocytes was found in DENV2-exposed cells; however, this difference was not statistically significant compared to mock-infected cells. The functions and phenotypes of the three types of monocyte populations during ZIKV or DENV infection are not well understood. Therefore, we further studied their immune profile in more detail in uninfected and ZIKV- or DENV2-infected cultures. We found that all three subtypes upregulated CD169, CD38, CD40, CD86, CXCL10, IL-6, or TNF-α at either 24 or 48 hpi. In general, we found higher levels of monocyte activation in ZIKV-infected cultures than in DENV2-infected cultures (Fig. 4C). Next, we compared the levels of expression of those activation markers across the different monocyte subtypes during infection by ZIKV or DENV2 or mock infection (Fig. 4D). We found that nonclassical monocytes expressed higher levels of CD86 than classical or intermediate monocytes independent of the stimuli. However, nonclassical monocytes expressed lower levels of CD38 in unstimulated conditions than the other subtypes and lower levels of CXCL10 or CXCL8 mostly in infected cultures (and in mock-infected cultures at 48 hpi). The rest of the markers analyzed (same as those for Fig. 4C) did not show statistical differences among the monocyte subtypes (data not shown). Overall, we found alterations in the proportion of nonclassical monocytes during ZIKV infection, probably driven by the modification of their surface CD14 and CD16 expression. Therefore, the three monocyte subtypes studied showed phenotypic activation during ZIKV and DENV infection, and nonclassical monocytes differed from the other subtypes in their levels of expression of CD86, CD38, CXCL10, and CXCL8.

### ZIKV and DENV2 induce activation of DCs and B cells.

**FIG 5:**
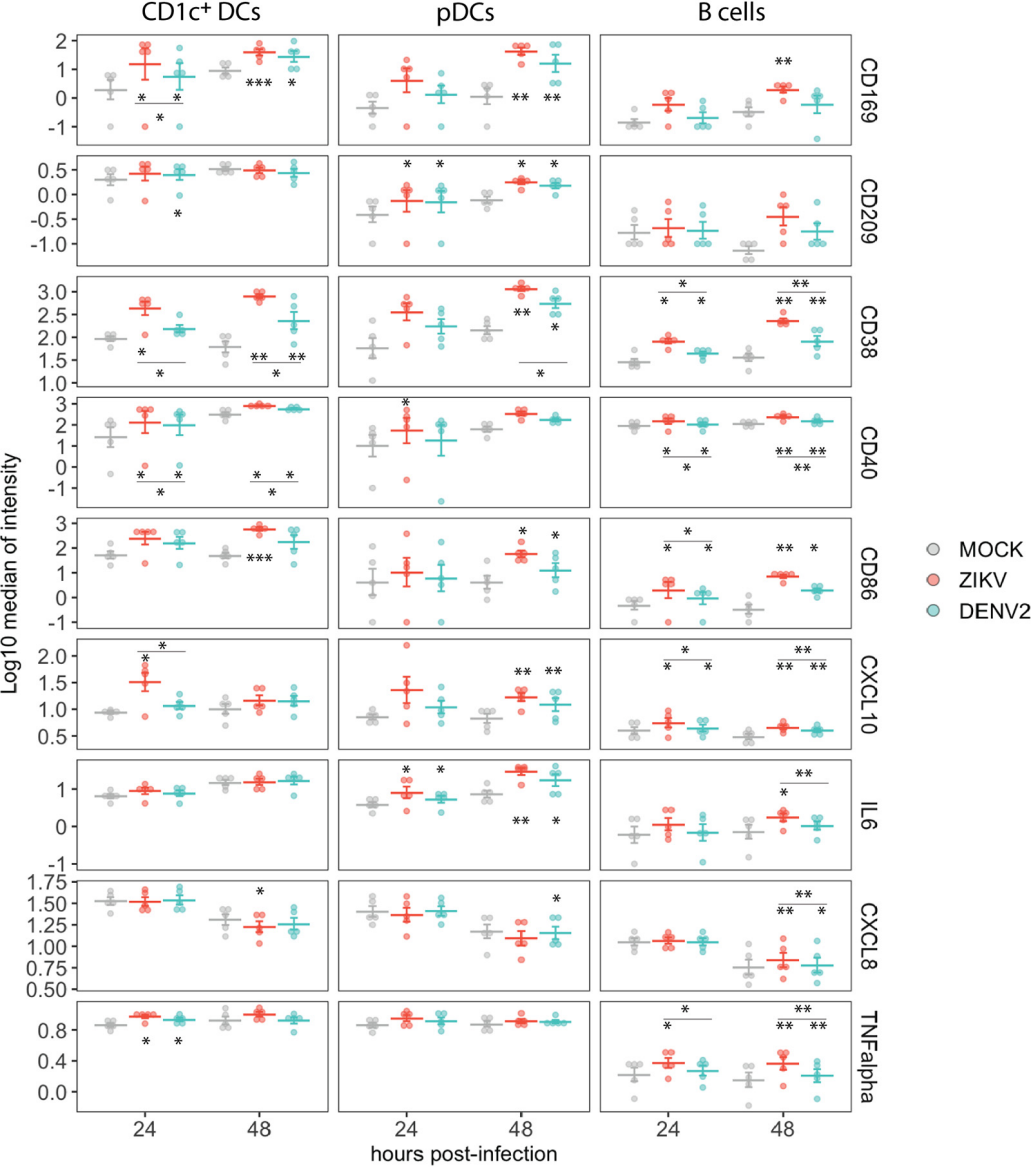
Expression of selected activation markers, cytokines, and chemokines in DCs and B cells in mock, ZIKV, and DENV2 *ex vivo*-infected PBMCs. Data from cells from 5 donors are shown. Means ± SEM are indicated. Stars without bars indicate statistically significant differences with mock treatment. ***, *P* < 0.001; **, *P* < 0.01; *, *P* < 0.05.

DCs are important cells during innate immune responses to virus infections, given their ability to sense virus pathogen-associated molecular patterns (PAMPs), phagocytose pathogens, and present antigens to B and T cells. Therefore, we analyzed the patterns of activation of DCs, specifically CD1c^+^ DCs (myeloid lineage) and pDCs (lymphoid lineage) during ZIKV and DENV2 *ex vivo* infections of PBMCs. As shown in Fig. 5, CD1c^+^ DCs showed great levels of upregulation of the markers CD169, CD38, CD40, and CD86. For cytokine production, we found significantly increased levels of TNF-α expression in ZIKV- and DENV2-infected cultures by 24 hpi and elevated levels of CXCL10 in ZIKV-infected cultures compared to mock-infected cultures. In the case of pDCs, we also observed increased expression of CD169, CD38, and CD86, and CD209 in DENV2- and ZIKV-infected cultures compared to mock-infected cultures. In addition, pDCs also upregulated the expression of IL-6 and CXCL8 during infection by either ZIKV or DENV compared to pDCs in mock-infected samples.

The clustering analysis in Fig. 1 showed phenotypic changes in B cells as well. Therefore, we performed a more detailed evaluation of this cell population. The receptor CD38, indicative of activation of B cells (25), was upregulated in B cells in ZIKV- and DENV2-infected cultures at 24 and 48 hpi (Fig. 5) compared to mock-infected cultures. The markers CD38, CD40, and CD86 were also found to be upregulated in B cells in ZIKV- and DENV2-infected cultures compared to mock-infected cultures. In addition, increased expression of CXCL10, IL-6, CXCL8, and TNF-α in B cells was also detected during exposure to ZIKV- or DENV2-treated compared to mock-treated cells (Fig. 5).

### ZIKV and DENV2 induce release of cytokines and chemokines in PBMC cultures.

**FIG 6:**
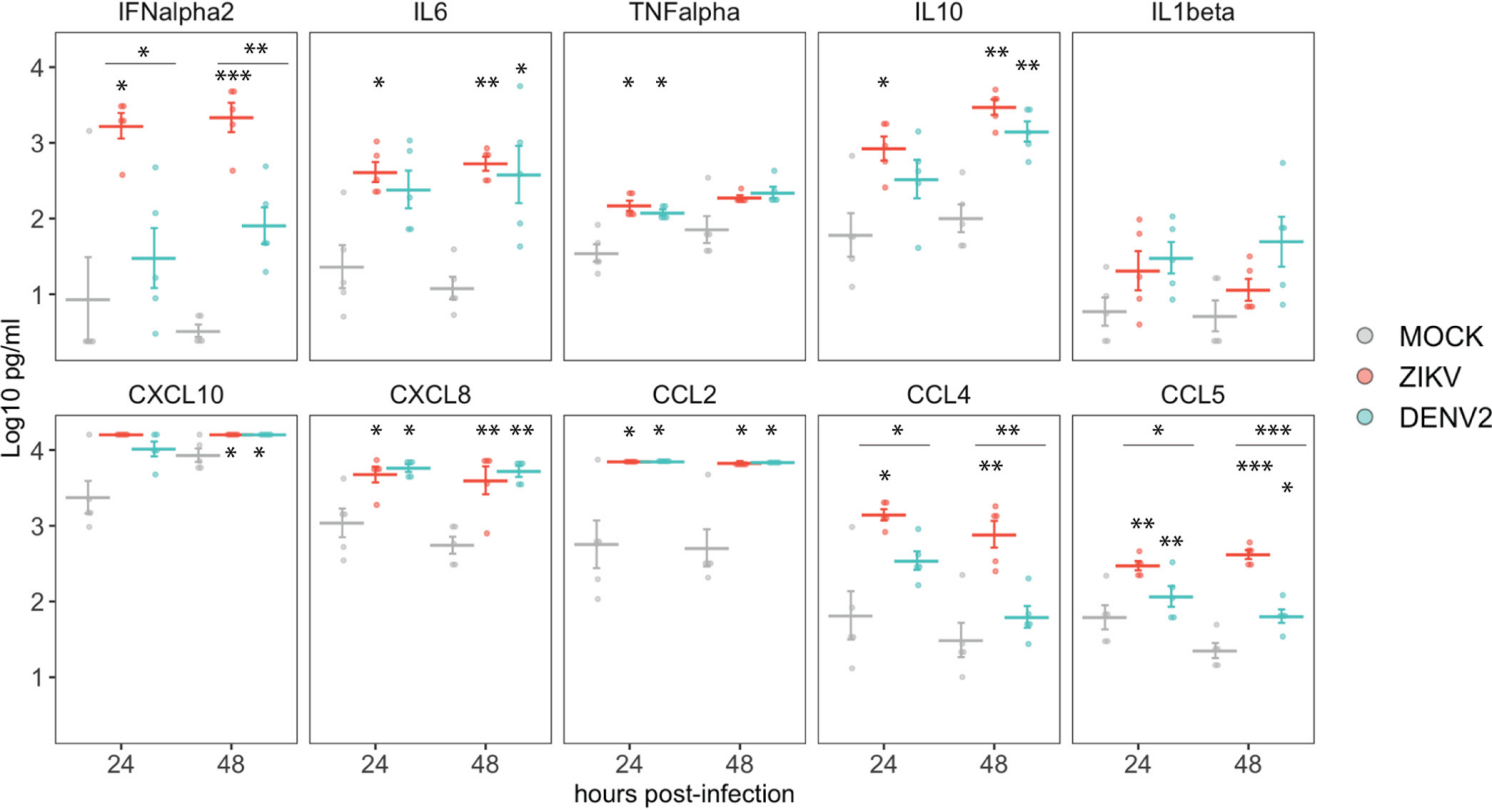
Cytokine production in supernatants of mock-, ZIKV-, and DENV2-exposed PBMCs in cultures quantified by Luminex assay. Data from cells from 5 donors are shown. Means ± SEM are indicated. Stars without bars indicate statistically significant differences with mock treatment. ***, *P* <0.001; **, *P* <0.01; *, *P* <0.05.

To evaluate the release of cytokines and chemokines by PBMC cultures mock treated or exposed to ZIKV and DENV2, we performed a Luminex assay on the supernatants of infected cultures. As shown in Fig. 6, increased release of interferon alpha (IFN-α), IL-6, TNF-α, IL-10, CXCL10, CXCL8, CCL2, CCL4, and CCL5 was detected in ZIKV- or DENV2-infected PBMC cultures compared to mock-infected samples (Fig. 6). Of note, ZIKV induced higher levels of IFN-α, CCL4, and CCL5 production in those cultures than DENV2.

### DENV2 stimulation of PBMCs induces distinct expression of cytokines and activation markers between bystander and infected myeloid APC.

**FIG 7:**
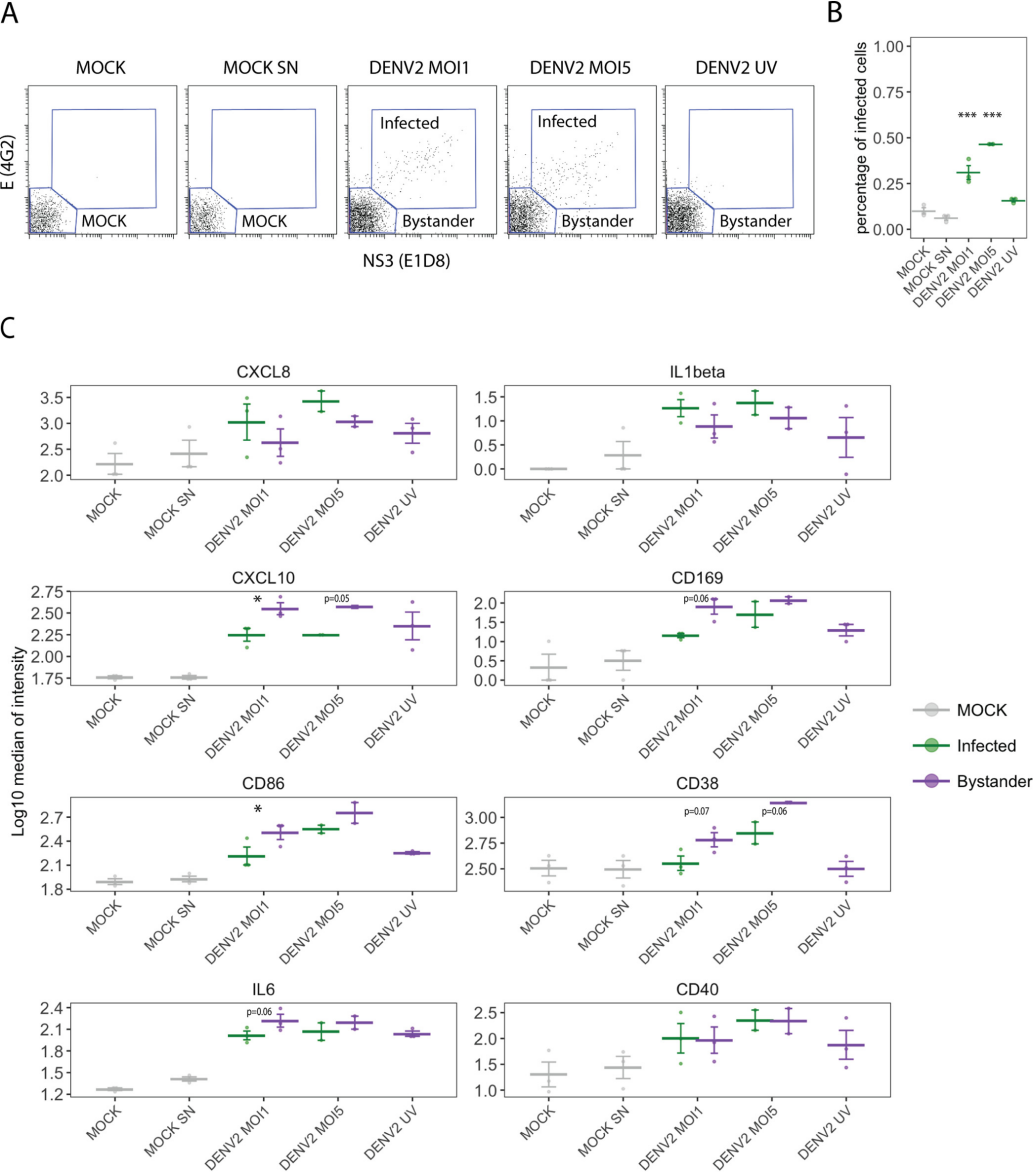
Analysis of the expression of activation markers in infected and bystander myeloid APC in response to DENV2 infection. PBMCs were incubated with DENV2 at an MOI of 1 or 5 or UV-inactivated DENV2 virus (MOI of 1) in the absence of any stimulus (mock) or in the presence of supernatant of C6/36 mosquito cells (mock SN). (A) Gating of infected cells (E- and NS3-positive cells) in myeloid APC population in a representative donor. (B) Percentage of infected cells in myeloid APC population. (C) Expression of markers in infected and bystander cells in infected cultures. Data from cells from 3 donors are shown (2 for DENV2 at an MOI of 5). Means ± SEM are indicated. Stars indicate statistically significant differences between infected and bystander cells. ***, *P* <0.001; **, *P* <0.01; *, *P* < 0.05. *P* values of >0.05 and <0.1 are also indicated with numeric values.

We previously found that DENV induces distinct phenotypic profiles in infected and bystander monocyte-derived dendritic cells (MDDC) (26). Given the low level of infection but marked activation in exposed cultures, our experiments suggest that most of the phenotypic changes are due to paracrine signaling. To better understand the interplay between infected and bystander cells in the PBMC system, we exposed PBMCs from 3 additional donors to two different MOIs of DENV2, 1 and 5, for 24 hpi, and analyzed the expression of the previously described markers using CyTOF. In addition, we added a condition in which PBMCs were treated with C6/36 supernatant from uninfected cells (mock SN) to eliminate the possibility of nonspecific activation due the presence of C6/36 cell products. To assess the need of active viral replication for the induction of activation markers, we also included PBMCs treated with UV irradiated virus stock (MOI of 1). Since we determined that myeloid APC are those with more phenotypic changes during virus exposure, we focused our analysis on this population. As shown in Fig. 7A, we gated infected cells, as determined by the presence of E and NS3 staining (4G2 and E1D8, respectively), and bystander cells (no expression of E or NS3), which were labeled as mock in the mock-infected cultures. As expected, no presence of E^+^ NS3^+^ cells was detected in the UV DENV2 exposed cultures. Next, we evaluated the expression of activation markers and cytokines in those populations and compared them between bystander and infected cells in infected cultures. While no significance was reached for CXCL8 and IL-1β, a trend toward higher expression in infected cell versus bystander cells was observed in PBMC-exposed cultures, similar to what we previously observed using MDDC (26). On the contrary, in the case of CXCL10, which is an interferon-stimulated gene (ISG) (27, 28), bystander cells showed higher expression than infected cells, consistent with the interferon signaling antagonism previously described for DENV (29–32). Interestingly, CD169, which is also an ISG (28, 33), had reduced expression in DENV2-infected cells compared to bystander cells, although this difference did not reach significance (*P*  = 0.06). Additional markers showed a similar pattern, specifically CD86, CD38, and IL-6, with reduced expression in infected cells compared to bystander cells. CD40 showed similar levels of expression in both bystander and infected cells. Some levels of activation were observed in the cultures exposed to UV DENV2 and, therefore, in the absence of active replication (CXCL10, CD169, CD86, and IL-6), although their levels of expression were lower than those found in infected cultures.

## DISCUSSION

We have applied the CyTOF technology to PBMCs from healthy donors to improve our understanding of the early innate immune responses to ZIKV and DENV infections induced in those cells. The use of *in vitro* and *ex vivo* systems to study the immune response to virus infection allows for controlled comparison of profiles induced by different viruses in clinically relevant primary cell types. One of the main advantages of these systems is that the innate immune profiles are compared in isogenic cells, reducing the effect of confounding variables such as genetic factors. In addition, as we control the timing of infection, early events that usually are not available when performing studies with patient or clinical samples can be captured using these methods. Our data showed that myeloid APC are the main cells in blood affected by exposure to ZIKV and DENV. The major components of myeloid APC in the blood are circulating monocytes and CD1c^+^ DCs. When we analyzed the phenotype of these two cell types, we observed a marked increase of the expression of activation markers and elevated production of CXCL10 and IL-6 compared to mock-treated PBMCs. In agreement with previous reports, we found that CD14^+^ monocytes are susceptible to infection by both ZIKV (16, 17) and DENV (19).

Myeloid APC such as monocytes and DCs are specialized phagocytic cells with an essential role in the initiation of the immune responses. These cells express high levels of cell membrane receptors, which are also considered attachment factors and are known to activate a variety of signaling pathways during the recognition and internalization of antigens (14). Viral glycoproteins have essential roles in the interaction with those receptors in the host cells. Flaviviruses such as ZIKV or DENV bind to glycosaminglycans (GAGs) as a first point of contact in the cell surface through GAG binding sites in their structural glycoproteins (34). Subsequent interactions with receptors then result in clathrin-mediated endocytosis. Some of the receptors that have been found to facilitate entry of flaviviruses are highly expressed in myeloid cell types, which might be related to their susceptibility to ZIKV and DENV infection. Some examples are CD14, C-type lectin DC-SIGN (10), and the mannose receptor (35, 36). This particular receptor composition of myeloid cells could facilitate the internalization of the virus and lead to cell activation by sensing of the viral RNA by intracellular pattern recognition receptors (PRRs) (37, 38).

In addition to classical activation markers such as CD86 and CD40, we observed great upregulation of CD169 and CD38 in monocytes, CD1c^+^ DCs, and pDCs after exposure to ZIKV or DENV compared to mock treatment. The receptor CD169, also known as Sialic Acid Binding Ig Like Lectin 1 (SIGLEC1), is highly expressed by specific subtypes of macrophages with a unique distribution in areas exposed to body fluids in secondary organs (39), which have been suggested to be specialized in processing antigen (40). Importantly, this receptor is not expressed in circulating immune cells in healthy individuals. The main role of CD169 is in cell-cell adhesion and interaction with pathogens (41). Interestingly, CD169 expression in immune cells is induced by type I IFN (28, 33), and it has been observed to be upregulated in monocytes in autoimmune diseases with a type I IFN signature, such as systemic lupus erythematosus and systemic sclerosis (42). Several studies also provide evidence of its involvement in virus infections. CD169 has been shown to be upregulated in monocytes from HIV patients (43), and an important role in DC-T cell transinfection of HIV has been described (44). In addition, it has been shown that rhinovirus induced the expression of CD169 in DCs (45). All these observations indicate that an upregulation of the CD169 receptor in circulating monocytes and DCs, otherwise absent in healthy individuals, is induced as part of a type I IFN-associated signature. This highlights a possible use of the receptor CD169 as a biomarker of a systemic type I IFN response during virus infections. Interestingly, the upregulation of CD169 in PBMCs from ZIKV (46) and DENV (19) has been identified in infected patients. Another important surface marker that we found to be upregulated in multiple cell types within PBMCs upon ZIKV infection is CD38. In contrast to CD169, CD38 is ubiquitously expressed in blood immune cells in healthy individuals, including B and plasma cells, T cells, NK cells, monocytes, and DCs (47). CD38 is considered a multifunctional protein, playing important roles in leukocyte migration and modulation of physiological functions through its enzymatic activity (48, 49). Interestingly, and consistent with our findings, a recent study identified CD38 as a marker of inflammation in human monocytes (50).

We also found changes in the PBMC monocyte subsets in ZIKV- and DENV-exposed cultures. The three main subsets of monocytes are defined by the expression of CD14 and CD16 surface molecules (24). Interestingly, we found a negative correlation between the expression of CD14 and multiple activation markers in myeloid APC when all conditions were combined. This indicates that the expression of CD14 is reduced as a consequence of the inflammatory environment, leading to differentiation to intermediate and/or nonclassical monocytes. In agreement with previous studies (16, 17), we observed an increase in the frequency of nonclassical monocytes in ZIKV-exposed cultures. Indeed, an expansion of intermediate and nonclassical monocytes has been found in multiple inflammatory diseases (51) as well as during bacterial and viral infection (24), supporting an effect of the inflammatory environment on the differentiation of circulating monocytes.

Our study allowed for the detailed characterization of the phenotype of these monocyte subtypes during ZIKV and DENV infection. There are conflicting data in the literature about the ability of each of the monocyte subsets to produce cytokines (52). Here, we found similar profiles of cytokine production for TNF-α and IL-6. However, nonclassical monocytes showed lower intracellular levels of the chemokines CXCL8 and CXCL10. A previous report by Cros et al. (53), which attributed a patrolling role in the blood vessels for nonclassical monocytes, found that this subset expressed similar levels of TNF-α and lower levels of CXCL8 than classical monocytes in response to multiple viruses, in line with our findings. We also detected higher levels of expression of CD86 in the nonclassical monocytes than the classical or intermediate monocytes, either with or without virus stimulation. However, while this is consistent with previous studies (52, 54), no differences between CD16^−^ or CD16^+^ monocytes to stimulate T cell proliferation has been identified (54). Further studies will clarify the functional effect of the differences on the level of expression of CD86 in these monocyte subsets.

We also analyzed the phenotype of myeloid APCs in infected and bystander cells from DENV2-exposed cultures. We found, similar to what we reported previously in the MDDC system, preferential expression of IL-1β and CXCL8 in infected cells compared to bystander cells (26). We also observed lower expression of CXCL10 in infected cells than in bystander cells, which is probably associated with the known roles of DENV2 in inhibiting STAT1 and STAT2 signaling (29–32). We found a similar pattern for the ISG CD169 as well as for CD86 and CD38. Interestingly, it has been reported that STAT2-deficient murine DCs have impaired upregulation of DC activation, particularly CD86 upregulation, upon treatment with type I IFN or TLR agonists (55). We have previously shown that DENV UV-inactivated virus does not induce stimulation of type I IFN or MDDC activation (56). However, in this study we observed upregulation of CXCL10, CD169, CD86, and IL-6 in UV DENV2-treated infected cultures, although to lower levels than in those exposed to live virus. Importantly, pDCs, which are a very important source of type I IFN and are part of the PBMC cultures, detect viral RNA (vRNA) through TLR7. It has been shown that DENV induces type I IFN and other cytokines in the absence of virus replication in pDCs, as opposed to myeloid DCs (57). Another study found that UV-irradiated DENV vRNA activates TLR7-mediated type I IFN expression in pDCs, although to reduced levels compared to non-UV irradiated vRNA (58). Therefore, it is possible that pDCs detect DENV2 in our PBMC cultures, leading to the global levels of activation that we observe in bystander cells.

Using a model of *ex vivo* PBMC infection and CyTOF technology, we have identified specific signatures for ZIKV and DENV infections in blood cells, characterized by an upregulation of activation markers mainly in myeloid cells but also in B cells and pDCs. Viral infection also resulted in changes in the frequencies of the monocyte subtypes, probably due to the exposure to an inflammatory environment, given the low frequencies of infected cells in the cultures. We also identified different phenotypes of those monocyte subpopulations. Among the proteins that showed increased expression upon infection, we found a clear upregulation of CD169, which, given its absent expression in blood cells in resting conditions, could be considered a marker for identification of viral infections in combination with other parameters in future studies. While the overall profile between ZIKV and DENV infection was similar, a stronger immune activation signature was found in ZIKV-infected cells compared to DENV-infected cells. These different phenotypes could be associated with the different abilities to infect the cells, replication efficiency, vRNA sensing, or their ability to antagonize the innate immune responses of these two viruses (30, 56, 59, 60). Importantly, the global phenotype of the *ex vivo*-infected PBMCs are in line with previous findings reported in ZIKV- and DENV-infected patients (16, 17, 19), such as upregulation of CD169 and CXCL10 in APC, and changes on monocyte phenotypes, validating the use of *ex vivo* infections to compare virus-induced phenotypes in blood cells.

## MATERIAL AND METHODS

### Virus stocks, isolation of PBMCs, and infections.

For ZIKV infections, we used the strain isolated in Puerto Rico in 2015 (GenBank accession number KU501215.1), PRVABC59 (61), which was kindly provided by Barbara W. Johnson (Centers for Disease Control and Prevention, Fort Collins, CO). The DENV strain used in this study belonged to serotype 2 (DENV2) and was isolated in Nicaragua in 2009 (GenBank accession number EU482690.1).

Virus stocks were prepared in C6/36 cells. Briefly, C6/36 cells are infected at a multiplicity of infection (MOI) of 0.01 for 7 days. The cell supernatants then were collected and stored at −80°C. Virus stock titers are determined by limiting-dilution plaque assays on BHK cells by following standard procedures. Inactivation of DENV2 virus stock was performed by exposing the supernatant of infected C6/36 cells during 10 min at a distance of 6 inches to UV germicidal irradiation.

PBMCs were isolated by Ficoll density gradient centrifugation (Histopaque; Sigma-Aldrich). A total of 10^6^ PBMCs were incubated with ZIKV or DENV2 at an MOI of 1 for 45 min at 37°C. The virus inoculum then was removed, and cells were washed with RPMI 1640 medium (Gibco) and incubated at a concentration of 10^6^ cells/ml in RPMI containing 10% fetal bovine serum (FBS) (HyClone Thermo Scientific), 2 mM l-glutamine, 1 mM sodium pyruvate, and 100 U/ml penicillin–100 μg/ml streptomycin (Gibco Invitrogen).

PBMCs were isolated from buffy coats provided by the New York Blood Center and were deidentified before being delivered to the investigators of this study. Therefore, this research is considered nonhuman subjects and does not require institutional review board approval.

### CyTOF.

Detailed information about the antibodies and reagents used for sample preparation are provided in Table S1 in the supplemental material. Antibodies were preconjugated from Fluidigm or conjugated and validated in-house using MaxPar X8 conjugation kits (Fluidigm Inc.). The monoclonal antibodies 4G2 and E1D8, specific for the E and NS3 proteins, respectively, were used for detection of viral proteins. At the specified times postinfection, we added 1 μM Rh103 nucleic acid intercalator to the cell culture medium and incubated for 20 min at 37°C. The cells then were washed with PBS containing 0.1% bovine serum albumin, blocked with human TruStain FcX (BioLegend), and incubated with a 0.1-μm-filtered cocktail of titrated MaxPar antibodies for cell surface markers for 20 min at 4°C. The cells were washed again, fixed, and barcoded using a Cell-ID 20-Plex Pd barcoding kit with a unique barcode ID assigned to each condition and time point. After barcoding, we pooled the samples to minimize staining variability, permeabilized using BD Cytofix/Cytoperm (BD Biosciences), and incubated with a 0.1-μm-filtered cocktail of MaxPar antibodies against intracellular antigens for 20 min at 4°C. Samples were washed again and incubated with 0.125 nM Ir nucleic acid intercalator to enable cell identification based on DNA content and stored in PBS containing 2% freshly diluted formaldehyde (Electron Microscopy Sciences) until acquisition. To minimize acquisition batch effects, samples collected at different time points were stored, combined, and acquired simultaneously as a single barcoded sample. Immediately prior to acquisition, the barcoded samples were washed in deionized water and resuspended at a concentration of approximately 600,000 cells/ml with EQ 4 element beads at a 1:20 dilution. Samples were acquired on a CyTOF2 using a SuperSampler fluidics system (Victorian Airships) at an event rate of <350 events/s. The CyTOF2 software was used to concatenate and normalize samples after data acquisition. The barcoded samples were deconvoluted using a Matlab-based debarcoding and doublet-filtering application (62), using a stringent Mahalanobis distance cutoff to ensure optimal barcode separation. The data were then uploaded to Cytobank version 5.2.0 (Cytobank Inc.) for analysis.

10.1128/mSphere.00505-21.3 TABLE S1 Detailed information of the antibodies and other reagents used for CyTOF sample preparation. Download Table S1, DOCX file, 0.02 MB. Copyright © 2021 Fenutria et al. 2021 Fenutria et al. https://creativecommons.org/licenses/by/4.0/ This content is distributed under the terms of the Creative Commons Attribution 4.0 International license.

### Luminex assay.

Analysis of cytokines and chemokines in the supernatants of infected cells was performed using the Milliplex MAP system (Millipore) according to the manufacturer’s instructions.

### Data analysis.

viSNE analysis and manual gating of the CyTOF data were performed using the Cytobank software. All the statistics derived from this analysis (percentage of infected cells and median of intensity for all the markers) were exported from Cytobank. R and R Studio were used to generate plots and for further statistical analysis. Pair-wise *t* test comparisons corrected using the Benjamini and Hochberg method were performed to identify statistically significant differences. Scripts for calculating the Jensen-Shannon (JS) divergence of the clusters identified in the viSNE plots are available at https://github.com/ismms-himc/cytutils.

### Data availability.

Data from CyTOF and luminex assays performed in this study have been deposited in the ImmPort database under the number SDY1530.
