# Supplementary material for: CyTOF Profiling of Zika and Dengue Virus-Infected Human Peripheral Blood Mononuclear Cells Identifies Phenotypic Signatures of Monotype Subsets and Upregulation of the Interferon-Inducible Protein CD169
Source: mSphere. 2021 Jun 23;6(3):e00505-21. doi: 10.1128/mSphere.00505-21 (PMC8265667; doi:10.1128/mSphere.00505-21)
Supplement: FIG S1 [file msphere.00505-21-sf001.pdf]

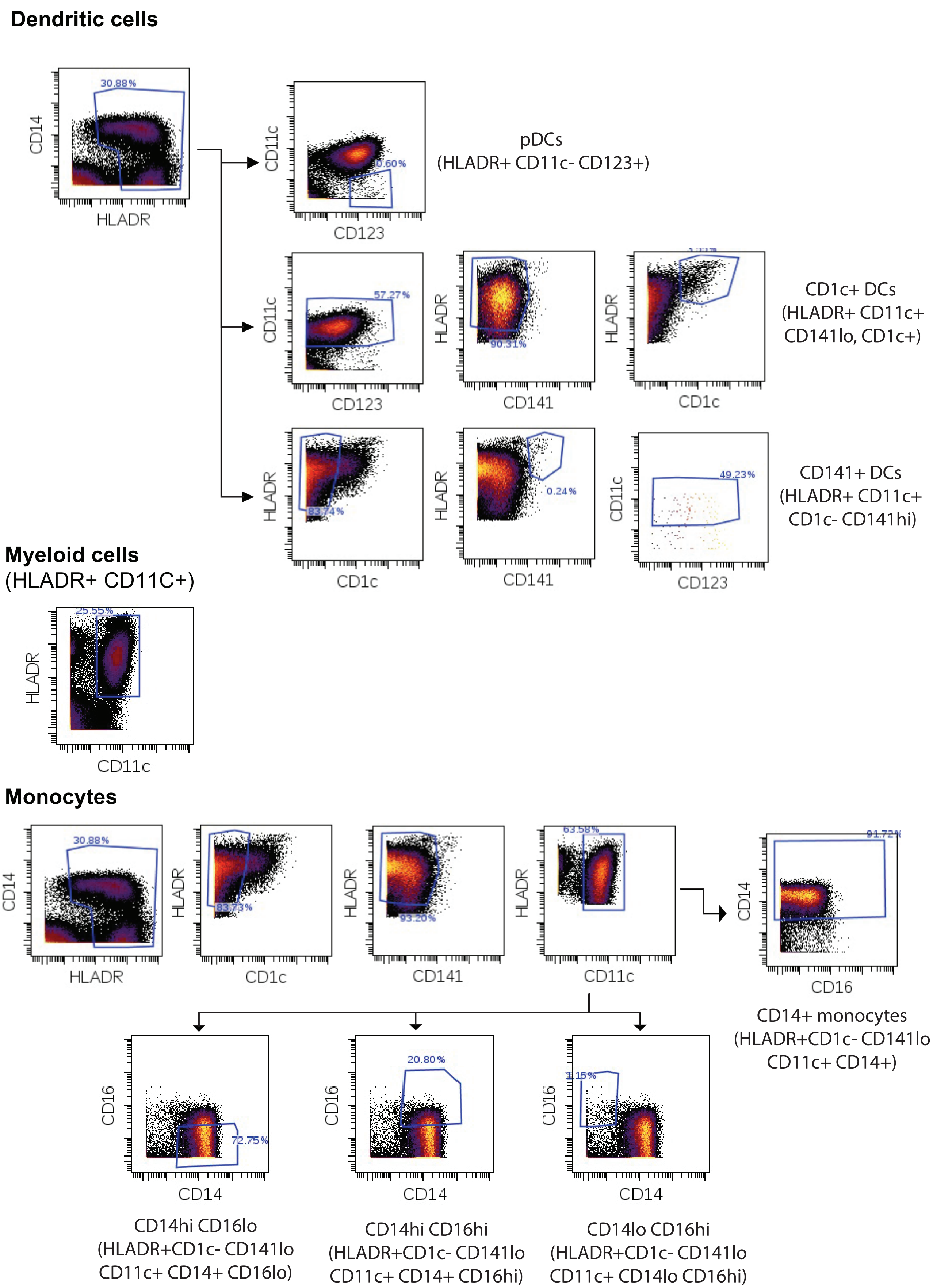

Supplementary Figure 1. Gating strategy for dendritic cells, myeloid cells, monocytes
