# Supplementary material for: CyTOF Profiling of Zika and Dengue Virus-Infected Human Peripheral Blood Mononuclear Cells Identifies Phenotypic Signatures of Monotype Subsets and Upregulation of the Interferon-Inducible Protein CD169
Source: mSphere. 2021 Jun 23;6(3):e00505-21. doi: 10.1128/mSphere.00505-21 (PMC8265667; doi:10.1128/mSphere.00505-21)
Supplement: TABLE S1 [file msphere.00505-21-st001.docx]

| **Reagent ID** | **Provider** | **Catalog Number** | **Analyte** | **Clone** | **Reporter** |
| --- | --- | --- | --- | --- | --- |
| Intercalator_Rh103 | Fluidigm | 201103A | Nucleic acid | N/A | 103Rh |
| Intercalator_Ir | Fluidigm | 201192B | Nucleic acid | N/A | 191/193Ir |
| Pd barcoding | Fluidigm | 201060 | Protein | N/A | 102-110Pd |
| CD16_148Nd | Biolegend | 302014 | CD16 | 3G8 | 148Nd |
| CD86_154Sm | Biolegend | 305410 | CD86 | IT2.2 | 154Sm |
| CD14_160Gd | Biolegend | 301810 | CD14 | M5E2 | 160Gd |
| CD40_164Dy | Biolegend | 334302 | CD40 | 5C3 | 164Dy |
| CCR7_167Er | Fluidigm | 3167009A | CCR7 | G043H7 | 167Er |
| CD209_172Yb | Biolegend | 330102 | CD209 | 9E9A8 | 172Yb |
| HLADR_174Yb | Biolegend | 307602 | HLADR | L243 | 174Yb |
| CD11b_209Bi | Fluidigm | 3209003B | CD11b | ICRF44 | 209Bi |
| IL1b_147Sm | Biolegend | 511602 | IL1B | H1b-98 | 147Sm |
| IL6_156Gd | Fluidigm | 3156011B | IL6 | MQ2-13A5 | 156Gd |
| DENV E_141Pr | ATCC and Biomatik |  | DENV E | D1-4G2-4-15 | 141Pr |
| IL8_173Yb | Biolegend | 511402 | IL8 | E8N1 | 173Yb |
| DENV NS3_175Lu | Eva Harris Lab (UC Berkeley) and Biomatik |  | DENV NS3 | E1D8 | 175Lu |
| CXCL10_176Yb | Biolegend | 519502 | IP-10 | J034D6 | 176Yb |
| CD57_113In | Biolegend | 322302 | CD57 | HCD57 | 113In |
| CD11c_115In | Biolegend | 301616 | CD11c | Bu15 | 115In |
| CD19_142Nd | Biolegend | 302202 | CD19 | HIB19 | 142Nd |
| CD45RA_143Nd | Biolegend | 304102 | CD45RA | HI100 | 143Nd |
| CD141_144Nd | Biolegend | 344102 | CD141 | M80 | 144Nd |
| CD4_145Nd | Biolegend | 300502 | CD4 | RPA-T4 | 145Nd |
| CD8_146Nd | Biolegend | 301002 | CD8 | RPA-T8 | 146Nd |
| CD127_149Sm | Biolegend | 351302 | CD127 | A019D5 | 149Sm |
| CD1c_150Nd | Biolegend | 331502 | BDCA1 | L161 | 150Nd |
| CD123_151Eu | Biolegend | 306002 | CD123 | 6H6 | 151Eu |
| CD66b_152Sm | Biolegend | 305102 | CD66b | G10F5 | 152Sm |
| TNFa_153Eu | Biolegend | 502941 | TNFA | Mab11 | 153Eu |
| CD27_155Gd | Biolegend | 302802 | CD27 | O323 | 155Gd |
| CD33_158Gd | Biolegend | 303302 | CD33 | WM53 | 158Gd |
| CD56_161Dy | BD Biosciences | 555513 | CD56 | B159 | 161Dy |
| CD169_162Dy | Biolegend | 346002 | CD169 | 7-239 | 162Dy |
| CD172a_b_163Dy | Biolegend | 323802 | CD172a_b | SIRPa/b | 163Dy |
| CD3_168Er | Biolegend | 300402 | CD3 | UCHT1 | 168Er |
| IFNa2b_169Tm | BD Biosciences | 551795 | IFNA2 | 7N4-1 | 169Tm |
| CD38_170Er | Biolegend | 356602 | CD38 | HB-7 | 170Er |
| CD161_171Yb | Biolegend | 339902 | CD161 | HP-3G10 | 171Yb |
| Axl_175Lu | R&D Systems | MAB154500 | Axl | 108724 | 175Lu |
| CD45_194Pt | Biolegend | 304002 | CD45 | HI30 | 194Pt |
| CD45_195Pt | Biolegend | 304002 | CD45 | HI30 | 195Pt |
| CD45_196Pt | Biolegend | 304002 | CD45 | HI30 | 196Pt |
| CD45_198Pt | Biolegend | 304002 | CD45 | HI30 | 198Pt |
